# Supplementary material for: Wolbachia reduces virus infection in a natural population of Drosophila
Source: Commun Biol. 2021 Nov 25;4:1327. doi: 10.1038/s42003-021-02838-z (PMC8617179; doi:10.1038/s42003-021-02838-z)
Supplement: Supplementary file 2 — Supplementary Information [file 42003_2021_2838_MOESM2_ESM.pdf]

Supplementary Information for

Wolbachia reduces virus infection in a natural population

Rodrigo Cogni[1,*], Shuai Dominique Ding[2,**], André C. Pimentel[1,**], Jonathan P. Day[2], Francis M. Jiggins[2, *]

[1]Department of Ecology, University of São Paulo, São Paulo, Brazil

[2] Department of Genetics, University of Cambridge, Cambridge, United Kingdom

* Correspondence: rcogni@usp.br and fmj1001@cam.ac.uk

** contributed equally

Supplementary Figure 1. Phylogenies of novel and known viruses. Maximum likelihood phylogenies were reconstructed from their predicted RDRP protein sequences. Additional taxa were included on the basis of the top DIAMOND blastx matches of *Drosophila* viruses queried against the NCBI non-redundant protein database (the host species was not used as a criterion for inclusion). Taxa in green and orange are viruses previously described as being associated with *D. melanogaster* or other species of *Drosophila* respectively. Taxa in red are new *D. melanogaster* associated viruses described in this study. Taxa in blue were associated with other species of arthropods.

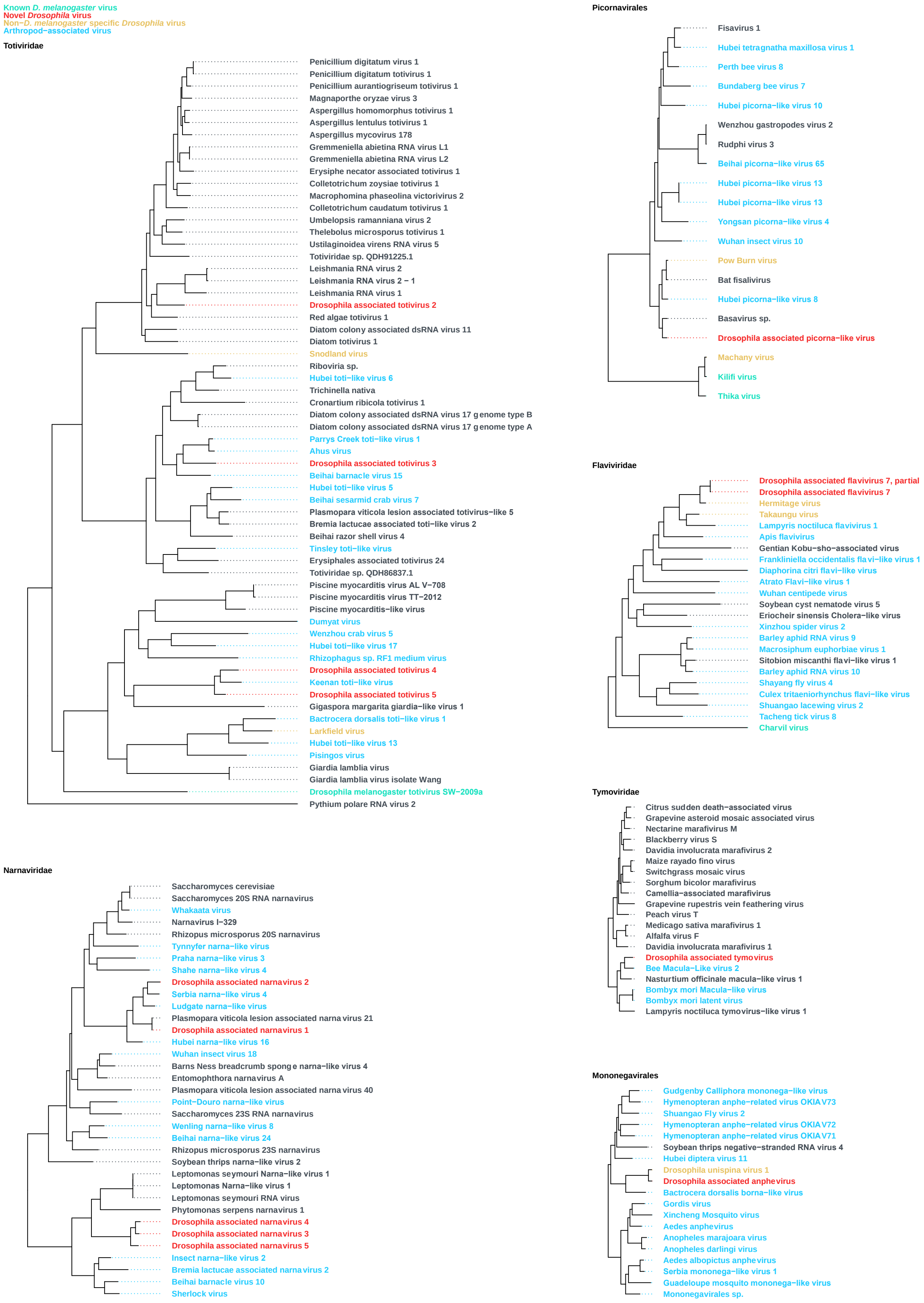
Known D. melanogaster virus
Novel Drosophila virus
Non−D. melanogaster specific Drosophila virus
Arthropod−associated virus
Totiviridae
Penicillium digitatum virus 1
Penicillium digitatum totivirus 1
Penicillium aurantiogriseum totivirus 1
Magnaporthe oryzae virus 3
Aspergillus homomorphus totivirus 1
Aspergillus lentulus totivirus 1
Aspergillus mycovirus 178
Gremmeniella abietina RNA virus L1
Gremmeniella abietina RNA virus L2
Erysiphe necator associated totivirus 1
Colletotrichum zoysiae totivirus 1
Macrophomina phaseolina victorivirus 2
Colletotrichum caudatum totivirus 1
Umbelopsis ramanniana virus 2
Thelebolus microsporus totivirus 1
Ustilaginoidea virens RNA virus 5
Totiviridae sp. QDH91225.1
Leishmania RNA virus 2
Leishmania RNA virus 2 − 1
Leishmania RNA virus 1
Drosophila associated totivirus 2
Red algae totivirus 1
Diatom colony associated dsRNA virus 11
Diatom totivirus 1
Snodland virus
Riboviria sp.
Hubei toti−like virus 6
Trichinella nativa
Cronartium ribicola totivirus 1
Diatom colony associated dsRNA virus 17 genome type B
Diatom colony associated dsRNA virus 17 genome type A
Parrys Creek toti−like virus 1
Ahus virus
Drosophila associated totivirus 3
Beihai barnacle virus 15
Hubei toti−like virus 5
Beihai sesarmid crab virus 7
Plasmopara viticola lesion associated totivirus−like 5
Bremia lactucae associated toti−like virus 2
Beihai razor shell virus 4
Tinsley toti−like virus
Erysiphales associated totivirus 24
Totiviridae sp. QDH86837.1
Piscine myocarditis virus AL V−708
Piscine myocarditis virus TT−2012
Piscine myocarditis−like virus
Dumyat virus
Wenzhou crab virus 5
Hubei toti−like virus 17
Rhizophagus sp. RF1 medium virus
Drosophila associated totivirus 4
Keenan toti−like virus
Drosophila associated totivirus 5
Gigaspora margarita giardia−like virus 1
Bactrocera dorsalis toti−like virus 1
Larkfield virus
Hubei toti−like virus 13
Pisingos virus
Giardia lamblia virus
Giardia lamblia virus isolate Wang
Drosophila melanogaster totivirus SW−2009a
Pythium polare RNA virus 2
Narnaviridae
Saccharomyces cerevisiae
Saccharomyces 20S RNA narnavirus
Whakaata virus
Narnavirus I−329
Rhizopus microsporus 20S narnavirus
Tynnyfer narna−like virus
Praha narna−like virus 3
Shahe narna−like virus 4
Drosophila associated narnavirus 2
Serbia narna−like virus 4
Ludgate narna−like virus
Plasmopara viticola lesion associated narnavirus 21
Drosophila associated narnavirus 1
Hubei narna−like virus 16
Wuhan insect virus 18
Barns Ness breadcrumb sponge narna−like virus 4
Entomophthora narnavirus A
Plasmopara viticola lesion associated narnavirus 40
Point−Douro narna−like virus
Saccharomyces 23S RNA narnavirus
Wenling narna−like virus 8
Beihai narna−like virus 24
Rhizopus microsporus 23S narnavirus
Soybean thrips narna−like virus 2
Leptomonas seymouri Narna−like virus 1
Leptomonas Narna−like virus 1
Leptomonas seymouri RNA virus
Phytomonas serpens narnavirus 1
Drosophila associated narnavirus 4
Drosophila associated narnavirus 3
Drosophila associated narnavirus 5
Insect narna−like virus 2
Bremia lactucae associated narnavirus 2
Beihai barnacle virus 10
Sherlock virus
Picornavirales
Fisavirus 1
Hubei tetragnatha maxillosa virus 1
Perth bee virus 8
Bundaberg bee virus 7
Hubei picorna−like virus 10
Wenzhou gastropodes virus 2
Rudphi virus 3
Beihai picorna−like virus 65
Hubei picorna−like virus 13
Hubei picorna−like virus 13
Yongsan picorna−like virus 4
Wuhan insect virus 10
Pow Burn virus
Bat fisalivirus
Hubei picorna−like virus 8
Basavirus sp.
Drosophila associated picorna−like virus
Machany virus
Kilifi virus
Thika virus
Flaviviridae
Drosophila associated flavivirus 7, partial
Drosophila associated flavivirus 7
Hermitage virus
Takaungu virus
Lampyris noctiluca flavivirus 1
Apis flavivirus
Gentian Kobu−sho−associated virus
Frankliniella occidentalis flavi−like virus 1
Diaphorina citri flavi−like virus
Atrato Flavi−like virus 1
Wuhan centipede virus
Soybean cyst nematode virus 5
Eriocheir sinensis Cholera−like virus
Xinzhou spider virus 2
Barley aphid RNA virus 9
Macrosiphum euphorbiae virus 1
Sitobion miscanthi flavi−like virus 1
Barley aphid RNA virus 10
Shayang fly virus 4
Culex tritaeniorhynchus flavi−like virus
Shuangao lacewing virus 2
Tacheng tick virus 8
Charvil virus
Tymoviridae
Citrus sudden death−associated virus
Grapevine asteroid mosaic associated virus
Nectarine marafivirus M
Blackberry virus S
Davidia involucrata marafivirus 2
Maize rayado fino virus
Switchgrass mosaic virus
Sorghum bicolor marafivirus
Camellia−associated marafivirus
Grapevine rupestris vein feathering virus
Peach virus T
Medicago sativa marafivirus 1
Alfalfa virus F
Davidia involucrata marafivirus 1
Drosophila associated tymovirus
Bee Macula−Like virus 2
Nasturtium officinale macula−like virus 1
Bombyx mori Macula−like virus
Bombyx mori latent virus
Lampyris noctiluca tymovirus−like virus 1
Mononegavirales
Gudgenby Calliphora mononega−like virus
Hymenopteran anphe−related virus OKIAV73
Shuangao Fly virus 2
Hymenopteran anphe−related virus OKIAV72
Hymenopteran anphe−related virus OKIAV71
Soybean thrips negative−stranded RNA virus 4
Hubei diptera virus 11
Drosophila unispina virus 1
Drosophila associated anphevirus
Bactrocera dorsalis borna−like virus
Gordis virus
Xincheng Mosquito virus
Aedes anphevirus
Anopheles marajoara virus
Anopheles darlingi virus
Aedes albopictus anphevirus
Serbia mononega−like virus 1
Guadeloupe mosquito mononega−like virus
Mononegavirales sp.

Supplementary Figure 2. Correlation of PCR assays and reads of mapped to different sequences. (A and B) Infection status (table) and viral load (plot, only infected samples) of two different segments of the vera virus genome (panel A) or regions of the Motts Mill genome (panel B). PCRs performed on individual flies. Viral load is measured by quantitative PCR relative to the Rpl32 mRNA. In Panel A the Y axis corresponds to primers that amplify the capsid protein gene (GenBank accession number: MT742172.1) and the X axis primers that amplify the RDRP gene (accession number: MT742171.1). (C) The number of reads mapping to a newly-discovered virus related to the Drosophila immigrans sigmavirus compared to the number of reads mapping to the *D. immigrans* cytochrome oxidase I gene (*COI*). (D) The number of reads mapping to Grom virus compared to reads mapping to Motts Mill virus. (E) Reads mapping to Machany virus compared to Kilifi virus. Read counts are normalised against the total number of mapped reads in the library. Pearson correlation coefficients are shown. In panels C-E each point represents a library made from a pool of flies

A

Vera Virus	Vera Virus PCR 1	
PCR2	Infected	Uninfected
Infected	265	12
Uninfected	2	716

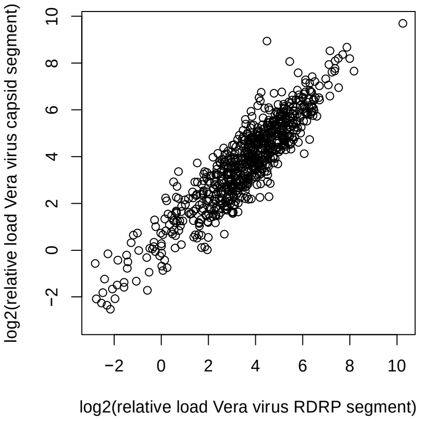

B

Motts Mill	Motts Mill PCR 1	
PCR2	Infected	Uninfected
Infected	763	2
Uninfected	7	178

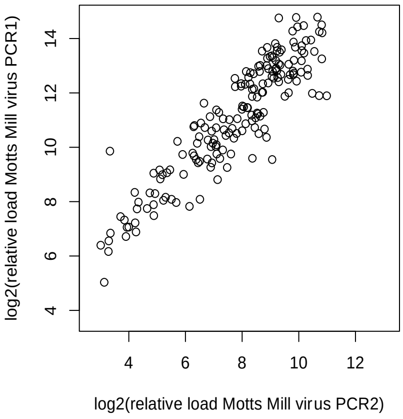

C

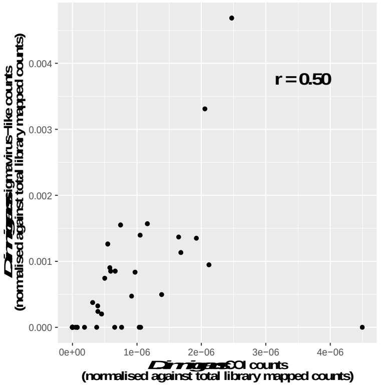

D

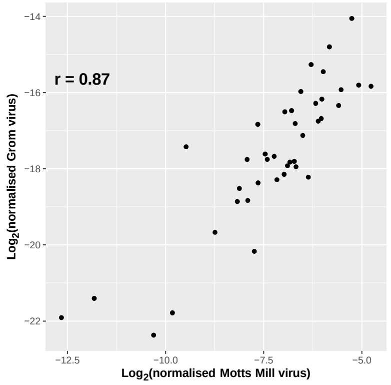

E

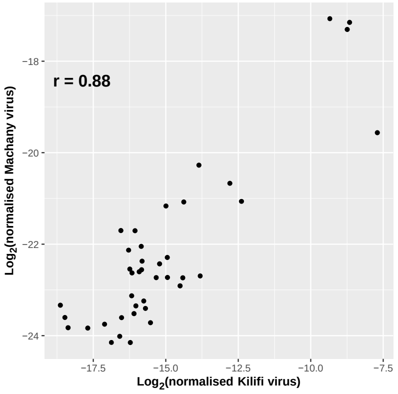

.

Supplementary Figure 3. The viral load in individual flies with or without *Wolbachia.* Vera and Motts Mill viruses were repeated with two sets of PCR primers. Only virus-infected flies are shown. P values are from a one-way ANOVA. Viral loads are expressed as viral RNA copy number relative to the *Drosophila* gene *Rpl32.* The data on Galbut virus is the same as that presented in the main text.

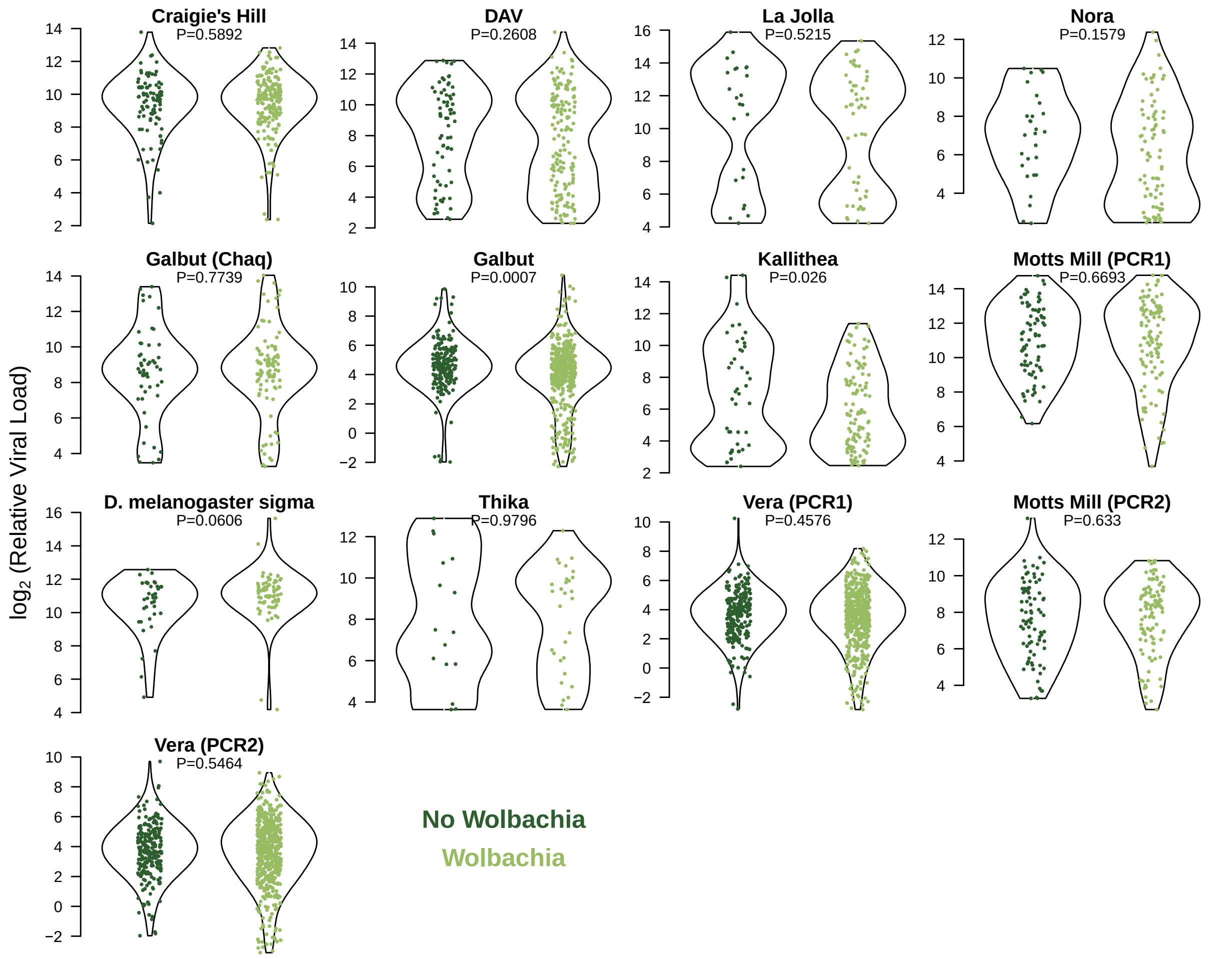

Supplementary Figure 4. Schematic of bioinformatics analysis.

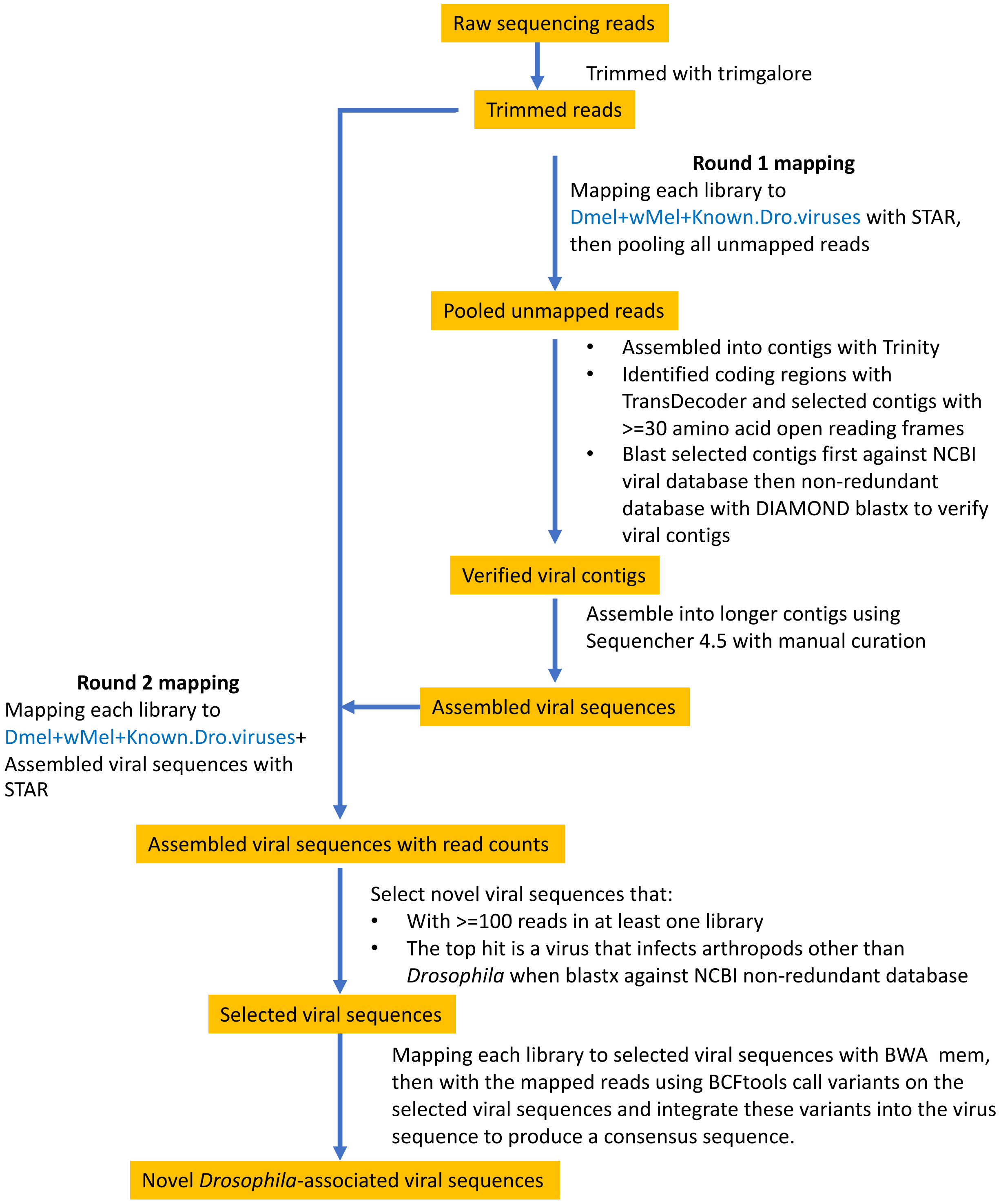

Legend

Dmel: *D. melanogaster* genome
wMel: *Wolbachia* strain *w*Mel genome
Known.Dro.viruses: all viral sequences in GenBank where the host is in the genus *Drosophila*

Supplementary Table 1. PCR primer sequences. Primer efficiency was estimated using a serial dilution of the template.

Virus	Primer Name	Sequence (5'-3')	Product Length (bp)	Primer Efficiency (%)
Galbut (Chaq)	Chaq2_Fw	TGAAATCACCATGCGTGTGC	105	105
	Chaq2_Rv	TCGTTAGCGCTCCACTTCTC		
Craigies Hill	C.Hill1_Fw	TAMCATCCAGCTGTTGCCAC	116	101
	C.Hill1_Rv	TTGCTGGTGAACCACCCATT		
DAV	DAV2_Fw	SATGGTTCAAGGACCCCGTC	83	99
	DAV2_Rv	TCTTGGGCATTGCACTGTAG		
D. melanogaster sigmavirus	Dromel.sigma3_Fw	AAAGATCTGGCAGTGAGCCC	80	102
	Dromel.sigma3_Rv	TCTCCTTACAGCCGAGATGA		
Vera virus	Vera_RDRP_Fw	AACCATTTCCGAGCGTAACCA	74	98
	Vera_RDRP_Rv	TTGATAGACGCTTGTGCCGC		
Galbut virus	Galbut1_Fw	CTCCGACACAAGTCGTCCAG	116	99
	Galbut1_Rv	GAGCAACTGCTTTCAGGCAC		
Motts Mill virus	MottsMill1_Fw	GAAGGTGGTTTTTGGGCGTT	118	92
	MottsMill1_Rv	AAGGCCAGATATAGCGCGAC		
Kallithea virus	Kallithea3_Fw	TTTCAGGGCACTCAGTAGCG	83	107
	Kallithea3_Rv	GCAACCTTTGCCACCTACAC		
La Jolla virus	LaJolla1_Fw	CGGACCAGAGTGTAGCCAAG	112	101
	LaJolla1_Rv	AGTGCCATCCAYCGATTTGT		
Motts Mill virus	MottsMill2_Fw	GAGACWTGGCAGTCTTTGGA	105	96
	MottsMill2_Rv	CCTATTTGCGGGGCATTTCG		
Nora virus	Nora1_Fw	AACGAGGAGCGATTGACGAG	85	103
	Nora1_Rv	TTGCTTCCAATTGCGCTGAC		
Thika virus	Thika3_Fw	CGTGGTGGTACAGGCAAAAC	113	105
	Thika3_Rv	TATGGGTCAGGTTCGGGTAA		
Vera virus	Vera_capsid_Fw	GAGGGGTAGAGGCGGTTTTC	96	97
	Vera_capsid_Rv	GGCATTGTTCCTCGCTCTCT		
