# Supplementary material for: Wolbachia reduces virus infection in a natural population of Drosophila
Source: Commun Biol. 2021 Nov 25;4:1327. doi: 10.1038/s42003-021-02838-z (PMC8617179; doi:10.1038/s42003-021-02838-z)
Supplement: Supplementary file 3 — Description of Additional Supplementary Files [file 42003_2021_2838_MOESM3_ESM.pdf]

Description of Additional Supplementary Files

File name: Supplementary Data 1

Description: Data behind figure 1. Read counts and prevalence for each virus.

File name: Supplementary Data 2

Description: Data behind figure 2A. Prevalence and credible intervals for each virus for Wolbachia-infected and Wolbachia-free flies.

File name: Supplementary Data 3

Description: Data behind figure 2B. Risk ratio and credible intervals for each virus.

File name: Supplementary Data 4

Description: Data behind figure 2C and supplemental figure 3. Viral load for each virus for Wolbachia-infected and Wolbachia-free flies.
