# Supplementary material for: Wolbachia reduces virus infection in a natural population of Drosophila
Source: Commun Biol. 2021 Nov 25;4:1327. doi: 10.1038/s42003-021-02838-z (PMC8617179; doi:10.1038/s42003-021-02838-z)
Supplement: Supplementary file 8 — Reporting Summary [file 42003_2021_2838_MOESM8_ESM.pdf]

nature research

Corresponding author(s): Rodrigo Cogni

Last updated by author(s): Aug 26, 2021

Reporting Summary

Nature Research wishes to improve the reproducibility of the work that we publish. This form provides structure for consistency and transparency in reporting. For further information on Nature Research policies, see our Editorial Policies and the Editorial Policy Checklist.

Statistics

For all statistical analyses, confirm that the following items are present in the figure legend, table legend, main text, or Methods section.

n/a	Confirmed	
☐	☒	The exact sample size (*n*) for each experimental group/condition, given as a discrete number and unit of measurement
☐	☒	A statement on whether measurements were taken from distinct samples or whether the same sample was measured repeatedly
☐	☒	The statistical test(s) used AND whether they are one- or two-sided<br>*Only common tests should be described solely by name; describe more complex techniques in the Methods section.*
☐	☒	A description of all covariates tested
☐	☒	A description of any assumptions or corrections, such as tests of normality and adjustment for multiple comparisons
☐	☒	A full description of the statistical parameters including central tendency (e.g. means) or other basic estimates (e.g. regression coefficient) AND variation (e.g. standard deviation) or associated estimates of uncertainty (e.g. confidence intervals)
☐	☒	For null hypothesis testing, the test statistic (e.g. F, t, r) with confidence intervals, effect sizes, degrees of freedom and P value noted<br>*Give P values as exact values whenever suitable.*
☒	☐	For Bayesian analysis, information on the choice of priors and Markov chain Monte Carlo settings
☒	☐	For hierarchical and complex designs, identification of the appropriate level for tests and full reporting of outcomes
☒	☐	Estimates of effect sizes (e.g. Cohen's *d*, Pearson's *r*), indicating how they were calculated

Our web collection on statistics for biologists contains articles on many of the points above.

Software and code

Policy information about availability of computer code

Data collection	No software was used for data collection.
Data analysis	STAR-2.6.0, bowtie2- v2.3.5.1, Trinity-v2.8.4, TransDecoder, DIAMOND blastx, Sequencher 4.5 , T-Coffee, PhyML. The code used for the bioinformatic analysis is available on the Github Repository at https://doi.org/10.5281/zenodo.5525968

For manuscripts utilizing custom algorithms or software that are central to the research but not yet described in published literature, software must be made available to editors and reviewers. We strongly encourage code deposition in a community repository (e.g. GitHub). See the Nature Research guidelines for submitting code & software for further information.

Data

Policy information about availability of data

All manuscripts must include a data availability statement. This statement should provide the following information, where applicable:

- Accession codes, unique identifiers, or web links for publicly available datasets
- A list of figures that have associated raw data
- A description of any restrictions on data availability

The RNAseq data has been submitted to the NCBI Sequence Read Archive under the BioProject number PRJNA728554. The assembled contigs of novel D. melanogaster associated viruses are available in GenBank (MZ852356 to MZ852369). The data underlying Figure 1 and 2 is available in Supplementary Data File 1 (figure 1), Supplementary Data File 2 (virus prevalence), Supplementary Data File 3 (risk ratios) and Supplementary Data File 4 (viral load).

Field-specific reporting

Please select the one below that is the best fit for your research. If you are not sure, read the appropriate sections before making your selection.

☐ Life sciences ☐ Behavioural & social sciences ☒ Ecological, evolutionary & environmental sciences

For a reference copy of the document with all sections, see nature.com/documents/nr-reporting-summary-flat.pdf

Ecological, evolutionary & environmental sciences study design

All studies must disclose on these points even when the disclosure is negative.

Study description	NA
Research sample	1014 D. melanogaster males collected at Lyman Orchards in Middlefield, CT, USA from the 4th to the 6th of September 2018
Sampling strategy	Sample size based on previous studies.
Data collection	RC collected the flies, JPD and RC extracted RNA and checked Wolbachia status. JPD prepared the libraries for sequencing. SDD performed the bioinformatic analyses, ACP carried out the qPCR for virus prevalence.
Timing and spatial scale	All samples were collected from the 4th to the 6th of September 2018.
Data exclusions	No data were excluded from the analysis.
Reproducibility	The virus prevalence for the two most common viruses were repeated with different primers and results were consistent.
Randomization	Not relevante, since flies with and without Wolbachia were collected randomly in the field.
Blinding	All RNAseq libraries were analyzed without knowing the Wolbachia status. All qPCR data on virus prevalence were obtained without knowing the Wolbachia status of each fly.

Did the study involve field work? ☒ Yes ☐ No

Field work, collection and transport

Field conditions	Sunny and warm days.
Location	Lyman Orchards in Middlefield, CT, USA. 41°29'47.2"N 72°43'53.1"W
Access & import/export	No permits necessary.
Disturbance	No disturbance.

Reporting for specific materials, systems and methods

We require information from authors about some types of materials, experimental systems and methods used in many studies. Here, indicate whether each material, system or method listed is relevant to your study. If you are not sure if a list item applies to your research, read the appropriate section before selecting a response.

Materials & experimental systems

n/a	Involved in the study	
☒	☐	Antibodies
☒	☐	Eukaryotic cell lines
☒	☐	Palaeontology and archaeology
☐	☒	Animals and other organisms
☒	☐	Human research participants
☒	☐	Clinical data
☒	☐	Dual use research of concern

Methods

n/a	Involved in the study	
☒	☐	ChIP-seq
☒	☐	Flow cytometry
☒	☐	MRI-based neuroimaging

Animals and other organisms

Policy information about studies involving animals; ARRIVE guidelines recommended for reporting animal research

Laboratory animals	Study did not involve laboratory animals.
Wild animals	We collected a total of 1014 D. melanogaster males by aspirating and netting over fermenting dropped peaches. Males were identified to species level and individually preserved in RNAlaterTM reagent a few hours after field collecting.
Field-collected samples	Study did not involve laboratory work on field collected animals.
Ethics oversight	No ethical or guidance was required because we studied Drosophila melanogaster.

Note that full information on the approval of the study protocol must also be provided in the manuscript.
